# Economic Evaluation of Environmental Health Interventions to Support Decision Making

**DOI:** 10.4137/EHI.S1152

**Published:** 2008-12-19

**Authors:** Guy Hutton

**Affiliations:** Water and Sanitation Program, World Bank.

## Abstract

Environmental burden of disease represents one quarter of overall disease burden, hence necessitating greater attention from decision makers both inside and outside the health sector. Economic evaluation techniques such as cost-effectiveness analysis and cost-benefit analysis provide key information to health decision makers on the efficiency of environmental health interventions, assisting them in choosing interventions which give the greatest social return on limited public budgets and private resources. The aim of this article is to review economic evaluation studies in three environmental health areas—water, sanitation, hygiene (WSH), vector control, and air pollution—and to critically examine the policy relevance and scientific quality of the studies for selecting and funding public programmers. A keyword search of Medline from 1990–2008 revealed 32 studies, and gathering of articles from other sources revealed a further 18 studies, giving a total of 50 economic evaluation studies (13 WSH interventions, 16 vector control and 21 air pollution). Overall, the economic evidence base on environmental health interventions remains relatively weak—too few studies per intervention, of variable scientific quality and from diverse locations which limits generalisability of findings. Importantly, there still exists a disconnect between economic research, decision making and programmer implementation. This can be explained by the lack of translation of research findings into accessible documentation for policy makers and limited relevance of research findings, and the often low importance of economic evidence in budgeting decisions. These findings underline the importance of involving policy makers in the defining of research agendas and commissioning of research, and improving the awareness of researchers of the policy environment into which their research feeds.

## Introduction

Economic evaluation is a technique which experienced significant growth since the 1970s following the wide adoption of cost-benefit analysis for development programmers of the World Bank, United Nations agencies and bilateral donors [1,2]. Economic considerations began to play a central role in the selection of development projects covering infrastructure projects, support to commercial enterprise development, and agriculture [3–7]. This trend also influenced decision making in the health sector, with cost-effectiveness analysis guidelines beginning to appear in the 1970s, and becoming formalized by the end of the 1980s [8–11]. Economic evaluation consequently became firmly established and registered a gradual growth of research studies and initiatives covering individual publications [12–14] as well as initiatives supporting sector-wide health decision making such as the Disease Control Priorities Project [15], the U.K. National Institute for Clinical Excellence, or the Oregon State priority setting exercise [16].

Given the focus of health sector interventions on health outcomes, the main tool of analysis in the health sector has been cost-effectiveness analysis (CEA), as opposed to cost-benefit analysis (CBA) which is a technique more suited to other development programmers [12,17,18]. Subsequently, a major area of research within CEA has been the development of generic health indices such as disability-adjusted life-years (DALY), healthy life years (HLY), and quality-adjusted life-years (QALY) [19]. The focus of health economic studies on disease outcomes only was considered justifiable given the ‘silo’ approach to government decision making that still characterizes the allocation of public funds between government ministries. Except for the impact of health improvements on labor productivity—which has been the focus of some research, most notably the Commission for Macroeconomics and Health [20]—the cross-sectoral aspects of health have largely been ignored by health systems research which grows out of a paradigm that focuses on identifying and treating disease.

Environmental health is one area where this paradigm does not easily apply. Environmental health interventions include safe drinking water, improved sanitation and hygiene practices, vector control, reduced exposure to air pollution, food safety and poison control, solid waste management, water hazards (e.g. drowning) and can also include traffic safety, environmental noise reduction, occupational health and safety, ultraviolet radiation, and building safety. A broad issue of increasing importance touching on most of these topics is climate change, which alters exposure to these risk factors. Disease burden due to environmental risk factors is estimated to account for 24% of global DALYs and 23% of global deaths [21].

In terms of economic analysis, environmental health interventions have been recognized to be somewhat different to curative care approaches, given they bring with them non-health benefits, and often involve the interventions (and hence budgets) of other line ministries [22–24]. For example, improved access to drinking water may involve time savings, household productive activities as well as avoided health costs [25]. In the case of environmental management for vector control, intervention measures either have a dual benefit (increasing both agricultural production and reducing vector-borne disease transmission) or can be implemented at zero costs within existing programmers [23]. Hence the multiple benefit nature of environmental health interventions requires the combined effort of several ministries, including for example ministries dealing with health, agriculture, rural development, land, environment/natural resources, water resources and construction/infrastructure. This fact raises the need for a broader set of outcomes included in cost-effectiveness analysis, or indeed a broader technique such as cost-benefit analysis, to respond to decision makers information needs. Hence, decision making has to be carried out jointly between ministries or at least in coordination.

But how much economic research has been done in the area of environmental health interventions? How available is it to policy makers? How is it presented and targeted? And how good quality is it? These are key questions for both researchers and research users to answer, given that much economic research may not reach its intended audience, and even if it did, it may not be properly used to improve decisions involving policies or programmer funding.

Therefore, the aim of this article is to review the economic evidence available to decision makers in three environmental health areas—water, sanitation, hygiene (WSH), vector control and air pollution—and to critically examine the policy relevance and scientific quality of the articles for selecting and funding public programmers. It is expected the findings of this paper will be helpful for other environmental health fields where the economic evaluation literature is less developed.

The paper is organized as follows. Section 2 describes the review methods. Section 3 assesses the economic evidence base for environmental health interventions and its accessibility through scientific and other papers. This assessment is followed by a look in Section 4 into the relevance of economic evidence for decision makers—in terms of what interventions are being compared, the type of economic analysis, its comparability and the generalisability of evidence across sub-national and national borders. Section 5 reviews the scientific quality of the literature, examining the study design and quality of data sources used in economice valuation and evaluation of uncertainty. Section 6 concludes, making recommendations for steps researchers should take to increase uptake of their research, and how decision makers can be supported to play their role in selecting interventions which are socially efficient and fair.

## Methods

The review is based on articles sourced from a keyword search of title, abstract and MESH term from PubMed covering English-language articles with abstracts and on human subjects for the years 1990 to October 2008. There were no geographical or article-type limitations set in the search criteria. The review focuses on three fields: water, sanitation, hygiene (WSH), vector control, and air pollution. These are the environmental health topics with currently the highest number of published economic studies from developing countries and represent a major share of environmental health burden [21]. The initial search combined full spelling of any of the different analyses (cost-effectiveness analysis, cost-benefit analysis, cost-minimization analysis, cost-utility analysis) with any of the following terms: malaria, lymphatic filariasis, chagas, dengue, vector control, air pollution, hygiene, sanitation, and water. Water fluoridation and iodization are excluded, as were indoor allergens.

Given the likelihood of important economic evaluation studies outside PubMed, an internet search and reference follow-up was performed to identify other published literature, UN reports and grey literature. Only full economic evaluations comparing costs and benefits are included in the results tables due to space limitations, with reference in the text to other selected studies evaluating either costs or economic benefits (e.g. willingness to pay).

## The Economic Evidence-base and its Accessibility

The keyword search in PubMed revealed a total of 1192 English-language studies, which after title review gave 52 studies, and a closer assessment of abstracts or papers left 32 CEA or CBA studies in the three environmental health fields. In addition, internet search and follow-up of other sources revealed the following additional studies: 2 book chapters, 8 peer-reviewed published journal articles outside PubMed, and 8 other reports. This gives a total of 50 economic evaluation studies reviewed in this paper in the fields of water, sanitation and hygiene (13 studies), vector control (16 studies) and air pollution (21 studies), presented in Tables 1, 2 and 3, respectively.

For WSH interventions, the 13 studies found are distributed between 2 for drinking water supply alone [26,27], 4 for sanitation alone [28–31], 2 for hygiene alone [32,33], and 5 for combined WSH interventions [25, 34–37]. Previous reviews of studies also summarize the economic evidence-base [38,39]. The evidence base is spread across the world, with several articles from Africa and Asia, and a handful of global or regional studies. The English language-bias of the search methods lead to some papers found in PubMed not being reviewed due to insufficient information, such as three relevant studies published in the Chinese language [40–42].

For vector control, the 16 studies found are distributed between 10 for malaria control [43–52], 3 for dengue [53–55], 2 for chagas disease [56,57], and 1 for lymphatic filariasis [58]. The global spread of these articles are related to disease incidence, with chagas disease receiving focus in Latin America, dengue in Cambodia and Cuba, and malaria control focused mainly in African and Asian countries.

For air pollution a total of 21 studies are presented, with 15 for outdoor air pollution [59–73] and 6 for indoor air pollution [36,74–78]. Previous reviews and global assessments summarize the economic evidence-base, and these include some previously unpublished papers [79,80]. In addition to full economic evaluations, several studies focus on health damage costs of outdoor air pollution or willingness to pay for air pollution abatement in developing [81–83] and OECD countries [84–88]. Studies on outdoor air pollution are mainly national level studies from OECD countries (U.S.A. Europe, Japan), Taiwan, former Soviet Union (Kazakhstan, Hungary) and city-level studies covering China, Brazil and Mexico. Indoor air pollution studies are mainly from Africa, or are of a regional or global nature. Unpublished agency reports which follow established methodologies are included due to lack of published studies in the field of indoor air pollution [75,76].

As well as publications reporting results from new studies, there are also reviews or syntheses of evidence, which serve as an important source of economic evidence, especially for policy makers who are constrained in accessing and absorbing primary research evidence. The Disease Control Priorities Project is an important example, which in 2006 went through a second and expanded edition, with several chapters covering environmental health interventions [89–92], and an overview chapter comparing cost-effectiveness analysis of a wide range of health interventions [93]. A second global project, the Copenhagen Consensus, in 2006 reviewed the cost-benefit evidence for a range of health and non-health intervention to assist global priority setting [94] and in 2009 will produce a further set of outputs.

The review has focused on identifying and presenting studies that have been through a process of external peer-review, which is expected to lead to a higher average quality of publications. A lot of CBA studies conducted to help donor agencies and development banks to select development projects have been excluded, given the lack of peer review or the tendency of these studies to compare a very limited range of project options based on donor or recipient preferences [95]. Hence most studies reported in this paper have been published in academic journals or books (42 studies: 32 journal articles from PubMed, 2 book chapters, and 8 other journal articles outside PubMed), while 8 studies are published reports from technical and donor agencies. However, it should be noted that peer-reviewed *journal* publications are not as easy to get hold of as they need to be subscribed to or involve a charge-per-view, unless they are open source and hence freely available on the internet.

In conclusion, despite established economic evaluation techniques being around for over four decades, the economic evaluation of environmental health interventions still appears to be in its infancy. For example, in a World Bank review in 2004, the economic assessment of health and other benefits of energy programs is cited as one of four unchartered areas [96]. For other environmental health areas, such as occupational health, cancer prevention and road traffic safety, an economic literature does exist, but this is almost exclusively from developed countries, and hence of limited relevance for developing countries, where health risks, technical intervention options and their costs and benefits are very different.

## Policy Relevance of the Economic Evidence-Base

Economic evidence serves a number of different but mutually-supporting purposes:

1. Advocacy—economic impacts or cost-benefit evidence can be used as stand-alone evidence to motivate decision makers to invest in environmental health interventions.
2. Resource allocation decisions—when faced with evidence on the health or economic return of different expenditure options, and their financial or economic costs, policy makers may increase budgets for interventions which have a higher economic efficiency. Also, government departments, project managers and development banks are interested in which interventions are most efficient for addressing a specific problem or contributing to a specific development outcome.
3. Decisions of policy itself—these policies may reflect laws, regulations or programmatic priorities, resulting directly or indirectly in more spending by the public sector and households, and stimulate provision through the commercial sector [97,98].

These three uses of economic evidence are relevant at several levels of the decision making ‘system’, from national government and donor level, down through different tiers of local government, to service providers themselves such as hospitals, private sector, NGOs and consumers or beneficiaries.

Faced with such a range of uses and users of economic evidence, it is hardly surprising that economic evaluation studies are so diverse, reflecting a range of viewpoints, selection of interventions and types of cost and benefit included. This raises the question of what, in fact, is good practice when it comes to defining an economic evaluation study from the policy angle [99].

### Selection of relevant type of analysis and outcome measures

Different types of economic analysis are relevant for different decision makers and interventions. Crudely speaking, CEA is most useful for decision making where a single sector has clear and sole responsibility for a particular intervention, or when one outcome dominates all others. In the health sector, CEA uses health outcomes such as cases or deaths averted. Cost-utility analysis is a subset of CEA, when health outcomes are converted to a health index such as DALYs. Conversely, CBA is useful for multiple sector decision making, or when several outcomes contribute to overall benefit, and where it is possible to aggregate the different impacts in monetary units. Hence other outcomes than health can be included such as reduced damage to crops and buildings, or less CO_2_ emissions, as in the case of outdoor air pollution (see Tables 1–3). Cost-minimization analysis (CMA) or least-cost analysis (LCA) are techniques used when interventions produce the same outcome, thus the analysis focuses on identifying the least cost method of achieving a target outcome.

For decision making purposes, the cost-effectiveness literature on environmental health interventions is part helpful, part confusing. Most studies express outcomes in terms of cost per case averted, cost per death averted and/or cost per DALY averted or QALY gained. However, some studies present other outcomes due to difficulties of estimating health impacts, such as cost per person covered or protected from vectors [44–46,52], or per breeding container reduced [53], or for hygiene education, cost per percent increase in knowledge [33]. One study measures cost and effluent quality for constructed wetlands versus waste stabilization ponds for wastewater treatment, comparing reduction in biological oxygen demand [31]. Such intervention-, disease- or environmental-specific outcomes makes any comparison of efficiency between interventions difficult if not impossible. Furthermore, there is lack of standard approach to valuation and inclusion of cost offsets (direct cost savings), which makes it important to understand what is contained in the cost-effectiveness ratio (CER).

Cost-benefit analysis, on the other hand, is designed so that comparisons can be made across any set of interventions, so long as outcomes can be monetized. Hence CBA is more useful than CEA to central ministries such as Treasury, Finance, Economics or Planning. On the other hand, some studies do not present a benefit-cost ratio, but instead an internal rate of return or a net present value, thus raising the question of which decision criterion should be used first, or how to balance different decision criteria. Furthermore, some analyses adopt the perspective of a government provider or the household unit [43], while others adopt a societal perspective and take a longer term viewpoint [45,58].

A further confusion arises when some studies present cost-effectiveness ratios, while others cost-benefit ratios. For example, control of chagas disease has a cost per QALY gained of US$2.3 for vector control alone in Latin America [57] while a second study finds an internal rate of return of 64% in Argentina [56]. The economic literature on WSH interventions also suffers the same divide between CEA and CBA, shown in Table 1, whereas economic studies on air pollution tend to favor cost-benefit analysis (Table 3).

### Inclusion of all new intervention options

Ideally, economic studies evaluate all potential intervention options, including the baseline (current intervention). The actual choice of options evaluated in any single study should take into consideration the relevance of each available option based on affordability, population acceptance, and technical feasibility considerations. This could, however, lead to unmanageably large and complex studies, given that most environmental health options have a large number of possible solutions. In modeling studies, more options can be considered, while for field trials usually 4–5 trial groups is the maximum.

As well as environmental solutions to environmental health problems—which are mainly preventive or promotive in nature—there is also a need to compare the efficiency of environmental interventions with curative interventions. This enables answering of controversial questions such as “is it cheaper to treat diarrhea than to prevent it?” Such a comparison can be made either as part of a single study (model or field trial), or the efficiency of environmental interventions from one study can be compared with other studies evaluating curative interventions.

In the case of water and sanitation interventions, few studies enable comparison of different solutions to the same environmental health problem. A good example is the global study on low cost solutions for improving drinking water quality, where 5 solutions are compared [26]. Another good example is the comparison of three sanitation options in an urban area of Indonesia [30]. On the other hand, some economic studies group technical options into a single intervention arm [27,29,32,34,36], thus only increasing understanding of overall cost-benefit (e.g. for advocacy or policy purposes) but not comparison between options for actual selection of technology.

Economic studies of vector control generally compare 2–3 of the main program options for vector control, such as insecticide treated nets (ITN) versus indoor residual spraying (IRS) [45,50,51], or vector control versus drug administration [47], while some compare single versus combined interventions (allowing assessment of marginal gains from adding interventions) [49,57,58]. These latter types of study are preferred as they provide more information to decision makers on the cost-effectiveness on individual as well as packaged options. For example, the incremental cost per QALY for adding potential new drug treatment on top of vector control is US$288, which is considerably greater than US$2.3 per QALY for vector control alone [57]. Several studies only evaluate a single option [43,48,54,56], thus rendering it difficult to make conclusions about the most efficient strategy. The inclusion of only a small number of technical options is partly explained by the tendency of evaluation studies to be based on actual vector programmers, thus limiting the number of options that can be compared using field data. One study with 5 options modeled (Sri Lanka) presents results in terms of cost per person protected [46], whereas another modeling study in Africa epidemiological regions D and E compares seven malaria control options separately and in packages, including IRS [49]. The latter study provides an example of comparison of average cost-effectiveness ratios of a package of malaria control interventions (e.g. US$32 per DALY averted in Africa D) with incremental cost-effectiveness ratio of adding IRS to the package (US$96 per DALY averted).

Economic studies on outdoor air pollution (OAP) tend to be the most complex in terms of policy options considered. In most cases, current standards or future legislation are examined for all their required actions leading to costs and benefits (mainly health benefits); hence reflecting more a type of project analysis than an academic study which characterizes the WSH, vector control and indoor air pollution studies. One OAP study examines emissions reductions from traditional brick kilns in the informal sector, examining four different strategies [60], while another compares three ways of retrofitting diesel vehicle exhausts in Mexico City [100]. Indoor air pollution (IAP) studies, on the other hand, tend to be limited in the options they evaluate, most of them choosing improved stove [75,76], or improved stove versus improved fuel use [74,77,78], and hence fall a long way short of evaluating all the potential options for reduction in exposure to indoor air pollution [90].

Other studies attempt to answer a different question. Rather than assessing the cost-effectiveness of averting a child death through an intervention grouping (such as WSH), Larsen models the cost of averting a child death in India, China and four global regions through the potentially most cost-effective and low cost interventions available: water, sanitation, immunization, female literacy and hygiene improvement. He shows that the greatest value-for-money is in immunization and hygiene interventions [101]. Likewise, Tan-Torres and Philips both compare different ways of averting child deaths with different health interventions [39,102]. Assuming scientific robustness, these types of studies are very informative for decision makers at higher levels of government (concerning allocation of national funds) as well as for micro-decision makers such as commune councils or non-governmental organizations.

### Comparison of findings with other evidence

Decision makers reading research findings of economic evaluation studies are often interested in how the findings compare with other studies to validate results or understand differences. The risks associated with using the wrong evidence are high. Hence researchers should assume that decision makers want to know if the study findings support or contradict the current accepted wisdom. Hence to contextualize the study results, authors should compare and contrast with previous research, explaining similarities as well as differences. In this regard, systematic review articles which compile data in easy-to-read format are valuable to policy makers. However, even review articles become outdated due to price inflation and the publication of new studies.

The reviewed studies are generally poor at comparing and contrasting their results with other research results. This is partly due to space restrictions of journals, but may also reflect researchers’ lack of contact with policy makers. While many articles broadly compare their results from other selected studies, few conduct a more systematic comparison and explore reasons for the differences.

As well as improving the economic evidence base, other non-economic evidence is often crucial in making a decision. What impact do interventions have on poverty and distribution of health or wealth? How acceptable are interventions to households and other beneficiaries, and what is their effective demand? What other financing mechanisms can be employed to assure the sustainability of the intervention? These questions are rarely, if ever, answered in most economic studies.

### Assessment of generalisability

Economic evaluation studies are often undertaken not just for the context in which they are conducted (e.g. a district or a country), but also to be indicative of the efficiency of the same development interventions in other settings. Hence, in order to be useful in other settings, an analysis of what factors or variables contribute to the observed results should be presented. This is best undertaken by those conducting the original study, and reported in the same publication. However, this is very rarely done in economic studies of environmental health interventions. Furthermore, to allow policy makers and researchers to better understand the potential similarity of findings in their own setting, a disaggregation of costs and benefits can be very useful. This includes separate reporting of physical quantities and prices which make up the cost and benefit figures. In some cases, a spreadsheet model can be provided to interested readers, thus enabling them to review the methods and to recalculate the results under different scenarios or price or quantity assumptions (see ‘analysis of uncertainty’ below). While journals are increasingly demanding access to the models on which results are based, the reviewed studies did not provide such access.

## Scientific Quality of the Economic Evidence-Base

A large number of guidelines are now available for the economic evaluation of health interventions, including general guidelines that cover all health interventions [103–105], and specific guidelines for vector control [106], water supply [107], and indoor air pollution [108,109]. All disease-specific guidelines generally reflect the standard economic evaluation methods outlined in health economic evaluation guidelines such as Drummond et al [8].

Despite the availability of these guidelines and gradual improvements in economic evaluation studies over time, the scientific quality of health economic evaluation studies from the developing world remains variable [13,14,110]. This section reviews how the environmental health economic evaluation literature performs in relation to key aspects of scientific quality, in addition to quality aspects covered in the policy section.

Almost all articles presented in Tables 1–3 provide reference to at least one of the foundation or spin-off economic evaluation guidelines; however, few articles adhere closely to all the recommendations outlined in the guidelines. For example, not all studies state clearly the specific research question and viewpoint from the outset, and instead must be inferred from the variables included and results presented.

### Inclusion of all relevant costs and impacts/benefits

Relevant costs and benefits include important and easily measurable ones, with a focus on those ones which are likely to be different between the intervention options to enable a choice. For important but hard to quantify costs or benefits (i.e. ‘intangibles’) either some further effort is required to quantify these variables, or instead a descriptive analysis is carried out, focusing on the expected difference between the options (e.g. acceptability). Tables 1 to 3 show the main benefits included in the reviewed studies. On the cost side, costs are usually all-inclusive covering capital or investment costs, operation and maintenance, and programmer costs related to initial intervention uptake and monitoring. There is some variation, however, in the inclusion of benefits. For WSH interventions (Table 1), CEA studies include only health impacts and some include cost offsets (i.e. cost savings from improved health) [26,32,43], while CBA studies tend to include cost offsets, productivity gains, and the value of saved lives (VOSL). One CBA study included only health benefits [30], while a non-English language CBA study from China includes the fertilizer value of human waste [40]. Health impacts in CEA and CBA studies tend to include only diarrheal disease, usually child cases, and focus on household benefits and ignore community effects.

For vector control, all reported studies except one are CEA studies, and hence only include health impacts, although roughly one half include cost offsets. No studies simultaneously examine the impact of vector control on more than one disease vector. Many studies examine the intervention impacts on children only, which is the most vulnerable group. Several CEA studies present CER using both gross intervention costs as well as net intervention costs (i.e. with health cost savings (cost offsets) subtracted from gross costs).

For air pollution, most studies are CBA studies; despite this, the majority of studies only consider health impacts, while some examine fuel savings, crop and material damage (2 OAP studies), impact on forest and greenhouse gases (4 IAP studies), and time savings from less fuel wood collection and cooking time (4 IAP studies).

Hence, it is clear that different sets of benefits are included within as well as between intervention areas, making difficult any comparison of comprehensive benefits. Also, the benefits of environmental health interventions are systematically underestimated due to omitted but potentially important additional health and non-health impacts.

### Assessment of causality of impact

Measuring the benefits of development interventions is a very challenging and sometimes controversial task, and is the object of volumes of scientific enquiry. Presentation of the health or economic benefits of development interventions forms the major argument and rationale for undertaking any type of programmer, and needs to be taken seriously. Therefore, study authors should clearly state the scientific methods used: when using secondary (published) evidence the best and most appropriate evidence should be used and referenced; for field studies, the most applicable technique for the study location and available funding should be used. In the latter case, randomized matched prospective studies are the ‘gold standard’—i.e. preferred—technique, but this is not always possible due to funding, time or ethical constraints in conducting a randomized trial.

The data sources used to estimate health effects vary between different environmental health interventions. Some interventions are more amenable to randomized studies such as hand washing campaigns, water quality interventions, and vector control (as well as other malaria control interventions such as ITNs). No economic evaluations of WSH interventions are based on their own randomized trial, but instead they draw on case control studies [29] or they use a modeling approach based on meta-analyses of health intervention studies [25,26,34].

Economic evaluations of vector control options most commonly draw on national programmers [43,48,54,56] which allow comparison either over time or between intervention and non-intervention areas which serve as control, or are based on pilot programmers with prospective cohort studies [58] or randomized trials [50,51].

For assessing the health impacts of air pollution, the time periods and scientific design are more demanding, hence most air pollution studies use models with dose-response relationships which are extracted from a small number of scientific studies. However, in many of the reviewed studies in all areas, models with data inputs from scientific studies are commonly used.

### Measurement of physical quantities and valuation of financial and economic impacts

In measuring the physical quantities of the costs and benefits of interventions, authors should state the sources of the data, the data collection technique, and the sampling approach and sample size. The next step is to convert physical quantities of cost or benefit to monetary units, which includes valuation in a common year, thus adjusting past costs upwards to the base year (e.g. by the rate of inflation) and adjusting future costs and benefits back to the base year (using an appropriate discount rate that reflects social time preference). The years in which cost data are presented in the reviewed studies is provided in column 3 of Tables 1–3. While naturally the year of cost data varies from study to study, most studies present costs in US Dollars, thus aiding comparability.

Where products and markets exist, the conversion to financial values is relatively straightforward [111]. However, for some benefits where no product yet exists, or where there is no market (e.g. the value of health improvements) or no single price (e.g. emissions reduction units), alternative methods need to be used. A range of alternative economic methods are available, such as contingent valuation, human capital, and hedonic pricing [4,6,112]. Quite a considerable literature exists testing and comparing the alternative methods; hence study authors should review which methods are most relevant to their setting, and clearly state and justify the selected method(s).

### Assessment of uncertainty

Given the above considerations on the scientific aspects and the many policy contexts in which decisions are being made, it is not surprising that there remains considerable uncertainty in the results generated by the reviewed economic evaluation studies. Indeed, it is not the task of the researcher to get rid of uncertainty, but instead to reduce it where possible and to clearly express it where it cannot be reduced. On the one hand, uncertainty can be *reduced* by using the appropriate scientific methods and data sources in a well constructed model or primary study. On the other hand, uncertainty can be better *expressed* using a variety of methods including sensitivity analysis (how much does the result change when one or more variables are changed?), threshold analysis (what value does an input variable need to take in order to change the decision?), and probabilistic sensitivity analysis, which provides confidence intervals—or at least a measure of distribution—on the base case results.

A significant number of the reviewed studies conduct sensitivity analysis, either one-way sensitivity analysis (e.g. water quality [27] and Chagas disease [56]) or multi-way sensitivity analysis (e.g. curbing air pollution in Shanghai [63]). However, very few studies present confidence intervals based on a rigorous multi-way probabilistic sensitivity analysis [60]. Hence, decision makers for environmental health interventions have no evidence—from a scientific base—on the distribution of cost-benefit or cost-effectiveness ratios. This is in fact a major failing of the reviewed studies.

## Conclusions

This review of 40 economic evaluation studies on three groupings of environmental health interventions—WSH, vector control and air pollution—generally suggests that these interventions are worthwhile from the perspective of society. The interventions register benefit-cost ratios and economic rates of return that appear highly attractive, and cost-effectiveness ratios which would rank high in the health sector’s priorities. Furthermore, many of the analyses include all relevant intervention costs but omit some benefits which would make the interventions even more attractive.

In reviewing the evidence from a decision maker’s perspective, however, some key pieces of information are missing. First, decision makers—whatever their level—need some basis for comparison. The different units of measurement and the small range of interventions evaluated in the different studies makes it hard to compare results and select a single intervention or mix of interventions based on the criterion of ‘efficiency’. Also, these studies would need to be compared with curative interventions, other environmental health interventions, and other development interventions, to enable a decision that will maximize return on public (and private) funds.

Second, decision makers are limited by poor access to the scientific literature where these studies are published, as well as in their understanding of technical fields which are removed from their everyday lives of bureaucratic systems, politicking and crisis management. They are also likely to feel overwhelmed by the length of publications and would have difficulty balancing opposing findings and interpreting minor details and qualifications stated in the various studies.

Third, the quality of economic evidence of environmental health interventions is variable. The majority of economic evaluation results are based on models combining evidence from a variety of sources, which reduces the value of the study findings among the potential promoters of economic evidence (scientists and policy analysts) as well as the decision makers. The high levels of uncertainty are not sufficiently explored or its effects quantitatively evaluated to assess how errors or variation might affect the decision.

Fourth, in drawing on the economic evidence base, the range of environmental health impacts and interventions to address them are limited by the available economic studies. For example, the interventions to reduce air pollution are limited mainly to anthromorphic emissions such as industry and vehicles (outdoor air pollution) and biomass burning (indoor air pollution). In fact, air pollution can originate from natural sources such as dust storms, which transport aeroallergens that affect humans.

Therefore, the following are recommended in relation to future studies conducting economic evaluation of environmental health interventions:

- Increase efforts to capture the broader benefits of environmental health interventions, especially where it may provide information on the potential willingness to pay of beneficiaries or donors. Where appropriate, researchers should design combined cost-effectiveness and cost-benefit analyses, and attempt to capture all the key outcomes of each type of analysis. Thus decision makers from different sectors will have a basis for collaboration, more sectors will be able to use the findings, and different studies and development interventions will be more comparable.
- Those undertaking economic evaluation should be pressured through the research funding, study design and peer review processes to increase the policy relevance and scientific quality of their research. This requires: ensuring all relevant interventions and important benefits are included; selecting the appropriate sources of evidence and filling evidence gaps; improving assessment of how uncertainty affects base case results; and clearly presenting in the published article all key aspects of the economic evaluation framework.
- Explore within the same studies a comparison of environmental health interventions—which are mainly preventive and promotive in nature—with curative health care options, to help to guide the focus of disease control programmers.
- Further work is needed to compile, synthesize and update economic evidence for health care decision making, including the environmental health field, so that all intervention sets can be compared.
- Researchers work with decision makers to increase uptake of research results, through a variety of means including: greater role of the decision maker in the research agenda; improved dissemination of key results in digestible format to decision makers; continued efforts to educate researchers in decision processes and decision makers in research methods; and to ensure economic evidence is interpreted in a broader technical and policy framework through consultation with other experts.

## Figures and Tables

**Table 1.** Water supply, sanitation and hygiene interventions—main features of reviewed economic evaluation studies.

**Studies (intervention options)**	**Country**	**Study type and year of cost data**	**Benefits included**	**Costs included**	**Key results**	**Reference and source**
**Water supply only**						
Household disinfection and storage for HIV people	Uganda (rural)	CEA; RCT; 2004	Health (diarrhea)	Capital, O&M	US$5.2 DCA US$1,252 DALY	[27]PM
Source-based protection Household chlorination Household filtration Household solar disinfection Household flocculation	Global (AFR-E and SEAR-D presented; AFR-E shown here)	CEA; Model; 2002	Health (diarrhea)	Construction, O&M, programmer	US$123 DALY US$53 DALY US$142 DALY US$61 DALY US$472 DALY	[26]PM
**Sanitation only**						
Constructed wetland Wastewater treatment plant	Italy (Venice)	CMA; programmer; 2005	Treatment efficacy	Capital, O&M (excluding land)	Wetland half to one eighth cost	[28]PM
Constructed wetlands Waste stabilization ponds	U.K. (urban)	CEA; CMA; programmer; 2005	Treatment efficacy	Capital, O&M (including land)	Stabilization pond one third to one seventh cost of wetland	[31]PM
Latrine improvement (construction or rehabilitation)	Afghanistan (Kabul)	CEA; case-control study; 1999	Child mortality (diarrhea only)	Provider and household	US$3,436 per death averted	[29]PM
DEWATS + EcoSan DEWATS + biogas STP	Indonesia (Surabaya)	CBA; model; 2001	User fees; health costs; productivity	Construction, O&M	BCR 1.1 BCR 0.92 BCR 0.66	[30]BC
**Health and hygiene education only**						
Health education for mothers	Burkina Faso (Bobo-Dioulasso)	CEA; programmer evaluation and model; 1999	Health (diarrhea children under 5)	Start-up/running costs to provider and household	US$51 DCA US$8 DCA including cost offsets	[32]PM
Chemotherapy months 0,18 Chemo. Months 0,6,12,18 Health education Option 2 and 3 combined	Bangladesh (rural)	CER; randomized intervention; 1995	Health (intestinal parasites)	Programmer costs	3% PR per US$1 2.5% PR per US$1 0.3% PR per US$1 1.1% PR per US$1	[33,113]PM
**Combined WSH interventions**						
Latrines and safe water ORS (moderate diarrhea) Treat severe diarrhea	Guinea	CEA; model; 1994	Health (diarrhea children under 5)	Programmer costs	US$343 death US$7 death US$74 death	[35]PM
Water improvement alone Water and sanitation Household water treatment	Global, regional (AFR-E presented here)	CBA; model; 2000	Health; VOSL; productivity, time savings	Capital, O&M costs	BCR 4.9 BCR 6.0 BCR 6.3	[25,114] PM, DR
Water and sanitation Household water treatment	Global, regional (AFR-E presented here)	CBA; model; 2000	Health, VOSL, productivity, time savings	Capital, O&M costs	US£534 DALY US$24 DALY	[34]PM
Integrated biogas, latrine and hygiene programmer	Africa-wide, Uganda, Rwanda, Ethiopia	CBA; model with field data; 2006	Fuel, health, productivity, VOSL, forest, greenhouse gases, time, lighting	Construction, O&M, programmer costs	BCR > 4.5 EIRR > 78%	[36]DR
SW added to existing HW HW and SW combined HW only SW only ORS	Global (generic)	CEA; model; 1996	Health (diarrhea children under 5)	Construction, O&M for HW; programmer costs for SW	US$20 DALY US$413 DALY US$1152 DALY US$44 DALY US$24 DALY	[37]PM

**Abbrevations:** ORS, oral rehydration salts; SW, software (hygiene and education); HW, hardware; CBA, cost-benefit analysis; CEA, cost-effectiveness analysis; CMA, cost-minimization analysis; RCT, randomized controlled trial; AFR-E, Africa WHO epidemiological strata E; SEAR-D, Southeast Asia WHO epidemiological strata D; WHO, World Health Organization; BCR, benefit-cost ratio; CER, cost-effectiveness ratio; CM, EIRR, economic internal rate of return; DEWATS, decentralized wastewater treatment; EcoSan, ecological sanitation; STP, off-site sewage treatment plant; DCA, diarrhea case averted; DALY, disability-adjusted life year (averted); PR, prevalence reduction.

**Notes:** Source: PM, PubMed (U.S. National Library of Medicine and National Institutes of Health); BC, book chapter; PR, other peer reviewed sources; DR, United Nations, donor, university or NGO report.

**Table 2.** Vector control interventions—main features of reviewed economic evaluation studies.

**Studies (intervention options)**	**Country**	**Study type and year of cost data**	**Benefits included**	**Costs included**	**Key results**	**Reference and source**
**Malaria control**						
IRS ITN Larviciding Water management Case treatment	Sri Lanka (North-Central province)	CEA; model; 1995	Health (malaria)	Annual programmer costs	Rs 116–158 PPP Rs 48 PPP Rs 27 PPP Rs 13 PPP Rs 112–153 PT	[46]PM
Integrated vector control with case treatment	Brazil (Amazon Basin)	CEA; time-series (1989 to 1996); 1996	Health (malaria); cost offsets	Annual programmer costs	US$69 DALY US$1,760 death US$1,517 death (cost offsets subtracted)	[43]PM
Comprehensive environmental management for vector control	Zambia	CEA; time-series (1930 to 1949); 1995	Health (malaria)	Annual programmer costs	US$858 death US$22 DALY	[48]PM
ITN IRS	Kenya (highlands)	CEA; programmer data; 2000	Health (malaria: people protected)	Annual programmer costs	US$2.34 PPP US$0.88 PPP	[45]PM
IRS in rural area IRS in urban area	Mozambique (Southern)	CEA; time-series (2000 to 2001); 2000	Health (malaria, age 2–14)	Annual programmer costs	US$29 per case prevented in all areas	[44]PM
ITN IRS	South Africa (KwaZulu Natal)	CEA; randomized trial; 2000	Health (malaria)	Annual programmer costs	ITN more costly and more effective than IRS: additional Rand 111 (US$18) PCA	[51]PM
IRS as part of mix of malaria control packages	Africa regions D and E (WHO)	CEA; model; 2000	Health (malaria) at 50,80,95% coverage	Full intervention costs (10 years)	Adding IRS to 3 other strategies costs US$96 DALY in Africa D region	[49]PM
ITN IRS	India (Gujarat state)	CEA; randomized trial; 1997–8	Health (malaria morbidity)	Annual programmer costs	Rs 1848 PCA Rs 3121 PCA	[50]PM
IRS Active case detection (ACD)	Nepal	CEA; programmer records; 1984	Cost offsets	Annual programmer costs	Rs 17–30 per capita Rs 3 per capita	[47]PM
ITN IRS	Vietnam (Hoa Binh Province)	CEA; survey and activity reports; 1996	Health (malaria: people protected)	Annual programmer costs	US$ 0.90 PPP US$ 0.47 PPP	[52]PM
**Control of other vectors**						
Vector control of dengue	Venezuela	CEA, CBA; model; 2005	Health	Annual programmer costs	US$122 DALY BCR 2.2	[55]PM
Annual larviciding campaigns to control dengue	Cambodia (Phnom Penh, Kandal)	CEA; programmer, health system records (2001 to 2005)	Health (dengue cases), cost offsets	Annual programmer and societal costs	US$313 DALY (provider); US$37 DALY (societal)	[54]PM
Dengue control (Aedes aegypti) Community vector control Vertical vector control	Cuba (Santiago de Cuba)	CEA; time series (2000 to 2002)	Health (dengue cases)	Capital, recurrent programmer costs, community costs	US$1,508 PBCR US$1,767 PBCR	[53]PM
Vector control of Chagas (American trypanosomiasis)	Argentina (Salta)	CBA; time series (1983 to 1992)	Health (chagas); cost offsets	Programmer and societal costs	IRR 64% NPV US$8 million	[56]PM
Chagas control Vector control alone Vector control + treatment	Latin America	CEA, CBA; model; 2000	Health (chagas); cost offsets	Programmer cost; assumed drug treatment cost	US$2.3 QALY US$3.4 QALY	[57]PM
Lymphatic filariasis MDA MDA + vector control	India (rural south India)	CEA; matched cohort (villages); 1997	Health (LF)	Programmer costs; villager time	US$1.8 per IMBA US$3.3 per IMBA	[58]PM

**Abbrevations:** ITN, insecticide-treated nets; IRS, indoor residual spraying; PPP, per person protected; PCA, per case averted; PT, per treatment; LF, lymphatic filariasis; MDA, mass drug administration; IMBA, infective mosquito bite averted; PBCR, per breeding container reduced. Other abbreviations: see Table 1.

**Table 3.** Reducing exposure to air pollution—main features of reviewed economic evaluation studies.

**Studies (intervention options)**	**Country**	**Study type and year of cost data**	**Benefits included**	**Costs included**	**Key results**	**Reference and source**
**Outdoor air pollution**						
Stabilized diesel/ethanol mixture in the bus and truck fleet	Brazil (Sao Paolo)	CBA; model; 2005	Job generation, potential carbon credits, health	Installation	NPV: US$2.8 m	[70]PM
Retrofitting diesel vehicles with Catalyzed diesel particulate filters Regenerating diesel particulate filters Diesel oxidation catalysts	Mexico (Mexico City)	CBA; model; 2000	Health (mortality)	Program (capital, recurrent)	For older/newer buses, cost per life saved: n.c./US$109,000 US$56,000/190,000 US$24,000/53,000	[71]PM
45 Federal Rules to reduce hazardous air pollutants	U.S.A	CBA; model; 2004	Health	Compliance and monitoring	BCR: 2.72–13.0	[69]DR
Clean Air Act	U.S.A	CBA; model (1990–2010); 1990	Health, crops, visibility	R&D, capital, operations and maintenance	BCR: 3.8	[68]DR
Acid Rain Program (Title IV of the 1990 Clean Air Act Amendments)	U.S.A	CBA; model; 2000	Health, visibility, fishing, ecosystem	Annualized costs until the year 2010	BCR: 40.7	[72]PM
Meet air quality targets	Europe	CBA; model; 2000	Health, productivity	Capital and recurrent	BCR: 6.0	[66]DR
Meet air quality standards	U.K	CBA; model; 2005	Health	Capital and recurrent	BCR 0.9–3.8	[67]DR
Substitute natural gas for coal	China (Beijing, Chongqing)	CBA; model; 1998	Health, fuel	Equipment and new fuel sources	IRR: 29% to 75%	[64]PR
Energy production emissions control Industrial sector emissions control	China (Shanghai)	CBA; model; 1998	Health, productivity	Regulations, facility closure, equipment	BCR: 1.1 BCR: 2.8	[63]PR
SO_2_ emissions control (3 policy eras)	Japan	CBA; model; 1993	Health, productivity	Investment, fuel conversion and running	BCR Phase 1. 5.39 BCR Phase 2. 1.18 BCR Phase 3. 0.41	[62]PR
NO_2_ emissions control (1973–94)	Japan (Tokyo)	CBA; model; 1995	Health, productivity	Capital and recurrent: industry and government	BCR: 6.1	[73]PM
Implementing national energy programmer	Hungary	CBA; model; 1994	Health, crops, materials	Investment and recurrent	BCR: 3.0–17.0	[59]PM
Emission reduction in oil extraction industry	Kazakhstan	CBA; case control; 2001	Health	Investment, maintenance	BCR: 5.7	[65]PR
Emissions reductions from traditional brick kilns Modified kilns Switch to natural gas Relocation of kilns No-burn days	Mexico (Juarez)	CBA; model; 1999	Health	Capital, operation and maintenance	US$29 m NPV US$29 m NPV US$16 m NPV US$1 m NPV	[60]PR
Fixed source emission reduction Vehicle emission reduction	Chile (Santiago)	CBA; model; 1996	Health	*(not stated)*	BCR: 2.4 BCR: 1.2 to 2.4	[61]PR
**Indoor air pollution**						
Improved stoves Clean fuel use (LPG)	Global, WHO sub-regions	CBA; model; 2005	Health, productivity, VOSL, fuel, forest, greenhouse gases, time	Investment, O&M, programmer	BCR negative BCR 1.5 to 21.2	[77]PR
Improved stoves Clean fuel use (LPG) Clean fuel use (kerosene)	Global, WHO sub-regions	CEA; model; 2000 (International $)	Health	Investment, O&M, programmer	US$500–7800 HLY US$1410–24100 HLY US$260–16200 HLY	[78]PR
Improved stoves	Malawi	CBA, CEA; field data; 2007	Fuel, forest, greenhouse gases	Stove and stove promotion	BCR: US$5.2 NPV: US$6.6 m	[76]DR
Improved stoves	Uganda	CBA, CEA; field data; 2007	Fuel, time, health, productivity, forests, soil, greenhouse gases	Stove and stove promotion	BCR: US$24.6 NPV: US$84 m	[75]DR
Improved stoves, LPG stoves, wall insulation or smoke hoods	Kenya, Nepal, Sudan	CBA; field data/model; 2007	Fuel, health, productivity, time	Societal investment and recurrent costs	BCR: 1.4 to 21.4 IRR: 19% to 429%	[74]BC
Integrated biogas, latrine and hygiene programmer (from Table 1)	SSA, Uganda, Rwanda, Ethiopia	CBA; model with field data; 2006	Fuel, health, productivity, VOSL, forest, greenhouse gases, time, lighting	Construction, O&M, programmer costs	BCR > 4.5 EIRR > 78%	[36]DR

**Abbrevations:** LPG, liquefied petroleum gas; HLY, healthy life year; SSA, sub-Saharan Africa; R&D, research and development; n.c., not calculated; m, million; Other abbreviations: see Table 1.
